# Comparing the Efficacy of a Combination of Diffusion-Weighted Imaging and T2-STIR (Short Tau Inversion Recovery) Imaging With Contrast-Enhanced MRI in the Evaluation of Perianal Fistula

**DOI:** 10.7759/cureus.53485

**Published:** 2024-02-03

**Authors:** Pooja Aggarwal, Rajesh Malik, Radha Sarawagi, Aman Kumar

**Affiliations:** 1 Radiology, All India Institute of Medical Sciences, Bhopal, IND

**Keywords:** contrast enhanced mri, short tau inversion recovery, t2 weighted imaging, diffusion weighted imaging, perianal fistula

## Abstract

Background: Perianal fistula is clinically diagnosed and commonly characterized using magnetic resonance imaging (MRI). Diffusion-weighted imaging (DWI) and T2-weighted imaging are emerging techniques that can obviate the need for contrast injection in cases where contrast administration is not feasible or contraindicated. The main objective of our study was to compare the efficacy of the combination of DWI and T2 STIR (short tau inversion recovery) imaging with contrast-enhanced MRI for the diagnosis and characterization of perianal fistula.

Methods: Sixty-nine patients with clinical perianal fistula with at least one external opening were evaluated with DWI, T2 STIR, and contrast MRI. A comparative cross-sectional study was conducted in the Department of Radiodiagnosis and Imaging, All India Institute of Medical Sciences, Bhopal, India. The chi-square test was done to find the association between categorical variables. The Kappa test was done to estimate the agreement between two different tests in measuring the outcome. The validity of tests was measured using sensitivity, specificity, positive predictive value, negative predictive value, and accuracy.

Results: The combination of DWI and T2 STIR is equivalent to contrast-enhanced MRI in the evaluation of primary and complicated perianal fistula. The combination of DWI and T2 STIR is superior to DWI alone in the classification and characterization of perianal fistula. However, DWI is superior to T2 STIR in differentiating perianal inflammation with abscess from perianal inflammation without abscess and can be used as an alternative to post-contrast fat-suppressed T1-WI in the detection of perianal abscesses and disease activity.

Conclusion: DWI can be used as an adjunct to T2 STIR, and the combination of DWI and T2 STIR can replace the post-contrast fat-suppressed T1 MRI sequence in the classification and characterization of perianal fistula.

## Introduction

Perianal fistula is a pathological connection between the infected glands in the anal canal and the perineal skin. Occlusion and infection of anal glands in the intersphincteric plane result in cryptoglandular abscess [1], and drainage of these abscesses, either spontaneously or surgically, results in fistula formation. This theory of the occurrence of anal fistulae is known as the cryptoglandular hypothesis [2]. Young males are twice as commonly affected as females. The most common clinical symptom is perianal discharge, followed by pain [3].

Perianal fistulas are commonly classified based on their anatomical locations. The most widely used classification is the Parks Classification, which distinguishes four kinds of fistula: intersphincteric, trans-sphincteric, suprasphincteric, and extrasphincteric. Park’s classification did not take into account risk factors for failure after surgery. To overcome this, a recent study was conducted in the Colorectal Surgery Unit, Mansoura University Hospitals [4], which described a modification of the Parks classification of anal fistula and examined its predictive validity in the assessment of the outcome of anal fistula in terms of failure of healing and fecal incontinence. An anorectal fistula is a clinical diagnosis, but imaging is required to determine the course of the tract and characterize it. Many different imaging studies have been tried in the past, including endoanal ultrasound, CT pelvis, and CT-fistulography, but failed to give satisfactory results. The most recent advancement in the field of diagnostic radiology utilizes MRI as the preferred diagnostic tool for the preoperative evaluation of perianal fistulas as it demonstrates very fine details of the primary tract of fistula, secondary extensions, and abscesses that eventually help the surgeon to plan surgery better and reduce the post-surgical recurrence rate.

Diffusion-weighted imaging (DWI) has been increasingly used for the detection of perianal fistula [5], complicating abscesses as well as the assessment of disease activity. Diffusion-weighted MRI reflects the changes in water mobility (known as Brownian motion) that occur due to their interactions with cell membranes and altered tissue environment [6]. The degree of diffusion of water molecules in tissue is inversely correlated with its cellularity and the integrity of cell membranes. As inflammatory tissues have increased cellularity, they exhibit “restricted” diffusion of water molecules that is evident in the form of contrast between affected cells and surrounding tissue [7].

## Materials and methods

This observational study of comparative cross-sectional design was conducted at a tertiary care hospital after approval from the Institutional Human Ethics Committee (IHEC), All India Institute of Medical Sciences Bhopal (approval number: IHECPGRMD035). All patients above 18 years of age referred for an MRI study with clinical perianal fistula were included in the study. Written informed consent was obtained from all participants. Patients with a previous history of fistula surgery, contraindications for MRI, claustrophobic patients, patients who did not consent, and cases with non-interpretable images were excluded from the study.

MRI acquisition

MRI was performed using a 1.5T superconducting system and an eight-channel phased-array body multicoil at All India Institute of Medical Sciences, Bhopal, India. Patients were asked to fast for six hours before the examination. No other special patient preparation was required. No bowel preparation or catheterization of the anal canal or fistula was performed. MRI scans were taken in coronal, sagittal, and axial planes after the selection of the proper localizer and FOV (field of vision). Slices were taken with no overlapping cuts. Slice thickness was 3-5 mm according to the lesion. Plain and contrast studies were done in all patients, and gadolinium was injected for showing contrast enhancement on T1WI using a 20G intravenous catheter at a concentration of 0.1 mL/kg. The imaging characteristics of lesions were then recorded in all patients. The anal canal is tilted forward by approximately 45 degrees in the sagittal plane; thus, oblique axial images (orthogonal to the anal canal) and coronal images (parallel to the anal canal) were obtained. To get an overview of the pelvis for correct orientation with the length and axis of the anal canal, an initial sagittal fast spin-echo (FSE) T2-WI was taken in all patients.

The most appropriate protocol used at our institution for perianal fistulas consists of the following sequences: high-resolution T2 STIR (sagittal, coronal, and axial), high-resolution T2 (sagittal, coronal, and axial), axial T1, axial 2 D diffusion, precontrast FS T1 (axial, coronal), and postcontrast FS T1 (sagittal, coronal, and axial).

MRI interpretation

The perianal fistula was evaluated on T1WI, T2 STIR, DWI, and post-contrast fat-suppressed T1W sequences. The radiologist was blinded to any MRI findings but was provided with the clinical history of the patient. MRI images of all patients were analyzed in two separate reading sessions at a two-week interval each to prevent recall bias. During the first session, only DWI images were analyzed for visualization and assessment of the primary track, activity in the track, internal opening, secondary extension, and perianal inflammation (with and without abscess). In the second session, DWI and T2 STIR images were reviewed for the same MRI parameters. Finally, the findings were compared with post-contrast fat-suppressed T1-weighted images. A two-point scale was used to register the findings as follows: 0 = not visualized; 1 = visualized. For the activity of the fistula, active clinical finding (active pus discharge) was considered as the reference standard, and the diagnostic accuracy of DWI, T2 STIR, and contrast were compared. For the rest of the imaging parameters, contrast images were considered as the reference standard, and the diagnostic potential of DWI alone, T2 STIR alone, and the combination of DWI and T2 STIR were compared. DWI images with a b value of 800 s/mm^2^ were used. Diffusion restriction was considered when DWI displayed a hyperintense signal with a corresponding drop in apparent diffusion coefficient (ADC) (high signal intensity on DWI with no drop in ADC can be due to the T2-Shine through effect). The lesions were evaluated and classified according to St James’s University Hospital classification.

The MRI findings of the perianal fistula were evaluated for the primary fistula tract, internal opening of the fistula, secondary extension of the primary tract, and abscess formation.

Statistical analysis

Data were collected using a preformed patient case datasheet. Data were entered into MS Excel (Microsoft Corporation, Redmond, Washington) and analysis was conducted using IBM SPSS Statistics for Windows, Version 21 (Released 2012; IBM Corp., Armonk, New York). Data were presented as mean ± standard deviation for continuous variables and as percentages for categorical variables. The chi-square test was performed to find out the association between categorical variables. The Kappa test was conducted to estimate the agreement between two different tests in measuring the outcome. The validity of tests was measured using sensitivity, specificity, positive predictive value (PPV), negative predictive value (NPV), and accuracy. A P value of less than 0.05 was considered significant.

## Results

Demographic analysis

In our study, 69 patients met the inclusion criteria and were hence included. The ages of the patients ranged from 18 to 70 years, with the mean age and standard deviation being 40.3 ± 12.7 years. The majority of the patients were from the age group of 31-50 years. The study included 69 patients, of whom 59 were males and 10 were females. A total of 80 perianal fistulae were detected in these 69 patients. Out of these patients, 9 (13.00%) had more than one fistula. According to the St. James’s University Hospital classification, most of the perianal fistulas were Grade 1 (42.5%), followed by Grades 2 and 4 (both 21.3%), then Grade 3 (11.3%), and the least common being Grade V (3.8%) fistula and abscess (Tables 1, 2).

**Table 1:** St. James’s University Hospital MRI classification system.

Grade	Description
0	Normal appearance
1	Simple linear intersphincteric fistula
2	Intersphincteric fistula with intersphincteric abscess or secondary fistulous track
3	Trans-sphincteric fistula
4	Trans-sphincteric fistula with abscess or secondary track within the ischioanal or ischiorectal fossa
5	Supralevator and translevator disease

**Table 2:** Frequency of cases according to St. James's University Hospital classification.

St. James’s University Hospital classification	Frequency	Percentage
Grade 1	34	42.5
Grade 2	17	21.3
Grade 3	9	11.3
Grade 4	17	21.3
Grade 5	3	3.8
Total	80	100.0

Conventional MR imaging parameters

Based on active clinical pus discharge, 54 (67.5%) of the fistulas were active, and 26 (32.5%) were inactive. For the detection of these tracts, almost perfect agreement was found between active pus discharge and DWI (P < 0.001), active pus discharge and T2 STIR (P < 0.001), and active pus discharge and post-contrast fat-suppressed T1 WI (P = 0.039). DWI alone could correctly identify active tracts in most cases with an accuracy of 95%. Out of the 80 fistulas, all 80 primary tracts were detected on the combination of T2 STIR and DWI (sensitivity 100%), T2 STIR alone could identify 75 primary tracts and had better performance (sensitivity 93.75%) than DWI alone, which could detect only 51 primary tracts (sensitivity 63.75%).

Of the 80 primary fistulas, the internal opening could be noted in only 70 (87.5%) cases by post-contrast fat-suppressed T1-WI. For the detection of the internal opening, almost perfect agreement was found between the combination of DWI and T2 STIR and post-contrast fat-suppressed T1 WI (P < 0.001), substantial agreement was found between T2 STIR and post-contrast fat-suppressed T1 WI (P < 0.001), and only slight agreement was found between DWI and post-contrast fat-suppressed T1 WI (P= 0.011). In comparison to post-contrast fat-suppressed T1 WI, the ability to correctly identify the internal opening was maximum for the combined DWI and T2 STIR, followed by T2 STIR alone, and minimum for DWI alone.

For the detection of secondary ramifications, almost perfect agreement was found between the combination of DWI and T2 STIR and post-contrast fat-suppressed T1 WI (P < 0.001) and between T2 STIR alone and post-contrast fat-suppressed T1 WI (P < 0.001). Substantial agreement was found between DWI and post-contrast fat-suppressed T1 WI (P < 0.001). Therefore, for the detection of secondary ramifications and horseshoeing, the combination of DWI and T2 STIR was the most superior, followed by T2 STIR alone, followed by DWI alone.

A total of 30 fistulas were seen having perianal inflammation in the form of ill-defined heterogeneous enhancement. Out of a total of 16 abscesses identified on post-contrast fat-suppressed T1 images, 13 abscesses were identified on DWI alone, with an accuracy of 96.25%, sensitivity of 81.25% (95% CI: 54.35% to 95.95%), and specificity of 100% (95% CI: 94.40% to 100.00%). Almost perfect agreement was found between DWI and post-contrast fat-suppressed T1 WI (P < 0.001).

Table 3 shows the frequency of detection of perianal fistula characteristics by DWI, T2 STIR, and the combination of DWI and T2 STIR, and Table 4 shows the diagnostic accuracy of DWI, T2 STIR, and the combination of DWI and T2 STIR in the characterization of perianal fistula.

**Table 3:** Frequency of detection of perianal fistula characteristics by DWI, T2 STIR, and combination of DWI and T2 STIR. DWI: diffusion-weighted imaging, STIR: short tau inversion recovery.

	Contrast	DWI alone	T2 STIR alone	Combination of DWI and T2 STIR
Activity in tract	54	50	50	50
Primary tract	80	51	75	80
Internal opening	70	29	62	67
Ramifications	30	19	26	30
Abscess	16	13	12	16
Perianal inflammation	30	0	30	30

**Table 4:** Diagnostic accuracy of DWI, T2 STIR, and combination of DWI and T2 STIR in characterization of perianal fistula. CI: confidence interval, PPV: positive predictive value, NPV: negative predictive value, DWI: diffusion-weighted imaging, STIR: short tau inversion recovery

		95% CI	Sensitivity	Specificity	PPV	NPV	Accuracy
Activity in tract	DWI	0.423 to 0.651	92.59%	100.00%	100.00%	86.67%	95.00%
Internal opening	DWI	0.595 to 0.804	41.43%	100.00%	100.00%	19.61%	48.75%
	T2 STIR	0.867 to 0.982	88.57%	100.00%	100.00%	55.56%	90.00%
	DWI+T2 STIR	0.918 to 0.998	95.71%	100.00%	100.00%	76.92%	96.25%
Ramifications	DWI	0.714 to 0.894	63.33%	100.00%	100.00%	81.97%	86.25%
	T2 STIR	0.855 to 0.977	86.67%	100.00%	100.00%	92.59%	95.00%
	DWI+T2 STIR	0.955 to 1.000	100.00%	100.00%	100.00%	100.00%	100.00%
Abscess	DWI	0.820 to 0.960	81.25%	100.00%	100.00%	95.52%	96.25%
	T2 STIR	0.782 to 0.938	75.00%	100.00%	100.00%	94.12%	95.00%
	DWI+T2 STIR	0.955 to 1.000	100.00%	100.00%	100.00%	100.00%	100.00%
Perianal inflammation	T2 STIR	0.820 to 0.960	100.00%	100.00%	100.00%	100.00%	100.00%
	DWI+T2 STIR	0.820 to 0.960	100.00%	100.00%	100.00%	100.00%	100.00%

## Discussion

MR imaging of suspected perianal fistula is an established method for the diagnosis and characterization of fistulas. The standard MRI sequence includes anatomical sequences with pre- and post-contrast images. The principal role of MRI in the setting of perianal fistula is to identify the primary tract, its extensions, side branches, activity of the tract, associated perianal inflammation, and deep abscesses. Currently, MRI scans are performed using endoanal coils, phased array coils, or a combination of the two, which provides complementary benefits.

DWI is being studied by some researchers to compare its performance, visualization, and characterization of perianal fistula and abscesses. In our study, we have classified perianal fistulas using St. James University Hospital classification. This classification is considered more advanced compared to Park's classification because it takes into account the primary fistulous tract as well as its complications, including the secondary extensions and abscesses [8]. Therefore, it provides a better correlation with surgical findings [9]. In our study, Grade 1 fistula was the most commonly recognized (42.5%), while complicated fistulae (grades 2 and 4) represented 21.3% each of the fistulae examined. Grade 3 fistulas constituted 11.3% of the fistulas, and the minimum number of cases had Grade 5 fistulas (3.8%).

Many researchers have claimed that T2 STIR sequences can be considered a good alternative to the post-contrast T1 sequence for identification and characterization of primary and complicated fistulas [10]. However, various other recent studies comparing multiple different sequences suggested that the T2 STIR sequence alone cannot completely replace post-contrast study due to its many limitations [11,12]. One major limitation of T2 WI is that edema displays high signal intensity, and it may be seen even in the fibrous area. On T2-weighted images, the fistula tract appears hyperintense since it is full of pus and granulation tissues [13]. These hyperintense signal changes may extend outside the fistula tract as surrounding soft tissue inflammation. As a result, both the fistula and surrounding soft tissue are similarly visualized with hyperintense signals on fat-suppressed T2-weighted images, which therefore is not able to differentiate between the two. This can be overcome by the use of DWI, which shows diffusion restriction in active fistulous tracts. While on post-contrast fat-suppressed T1-weighted images, diffuse enhancement is seen in the active granulation tissue lying on the edges of the fistula, and no enhancement is seen in its lumen containing fluid that shows hypointense signals. Inflammation surrounding the fistulous tract also enhances diffusely and heterogeneously [14]. Another limitation is that STIR images demonstrate high signal intensity in both abscess/fluid within a fistula tract and the granulation tissue, thus failing to differentiate between the two. While post-contrast images will demonstrate rim enhancement in abscesses and diffuse enhancement in granulation tissue [12].

In the current study, we also found that T2 STIR was not able to differentiate between abscess and perianal inflammation (without abscess). In these cases, DWI was found to be superior to T2 STIR in differentiating perianal inflammation from abscess, as no diffusion restriction was seen where perianal inflammation was present without abscess. Recent studies have suggested promising results with the use of DWI for perianal fistula cases complicated with abscess and thus may obviate the requirement for contrast administration [5]. These results were replicated in our study as we also found that DWI was equivalent in efficacy to post-contrast images in the detection of perianal fistulas complicated with acute abscesses. However, DWI had less sensitivity than T2 STIR alone in the identification of secondary extensions. Focusing only on the detection of the primary tract, some previous studies have suggested no significant statistical difference between DWI and T2-weighted images [15]. On the contrary, we found that the sensitivity of T2 STIR is significantly higher than DWI in the detection of the primary tract, whereas the combination of T2 STIR and DWI images had no significant difference in total accuracy compared to post-contrast T1 fat-suppressed images in detecting the primary tract.

T2 STIR alone could detect 93.8% of cases (only 5 cases could not be visualized on T2 STIR which were detected on post-contrast images), with DWI alone being the least sensitive in our study. We also found that the combination of DWI and T2 STIR was superior and comparable to post-contrast in detecting the internal opening, which could not identify the internal opening in only 3 cases. Correct assessment of the activity of the fistula tract is a crucial factor for anorectal fistulas in Crohn's disease patients, as these patients require frequent monitoring of the therapeutic response to their given medical treatment [16].

Active fistulous tracts are filled with pus and granulation tissue that shows enhancement on post-contrast images and hyperintense signals on T2 STIR images. No enhancement on post-contrast scans and loss of hyperintense signal on T2 STIR are markers of a healed fistulous tract. In our study, to look for the activity of the fistulous tract, we considered the clinical finding (active pus discharge) as the reference standard and compared the diagnostic accuracy of DWI and post-contrast images with it. We reached the conclusion that DWI alone could correctly identify active tracts in most cases with an accuracy of 95%. Post-contrast images could identify 100% active tracts; however, there were many false positive results with post-contrast images, thus reducing its overall accuracy. For the rest of the fistula parameters, the post-contrast scan was considered as a reference standard with which the performance and diagnostic accuracy of DWI alone, T2 STIR alone, and the combination of DWI and T2 STIR were compared. And we found that the combination of DWI and T2 STIR can give promising results and thus can replace post-contrast scans.

Our study has some limitations. First, we made contrast-enhanced T1-weighted images with fat suppression as the reference standard MR images, which may miss some fistulae and accompanying pathologies, especially the healing fistulae. So it may not be a reliable “reference standard” for perianal fistula disease in all cases. Second, most of our patients presented with low intersphincteric fistula; we, therefore, did not have enough cases with high fistulas. Finally, our results may vary from other authors who used higher b values for the DWI sequence (1000 s/mm²). 

**Figure 1:**
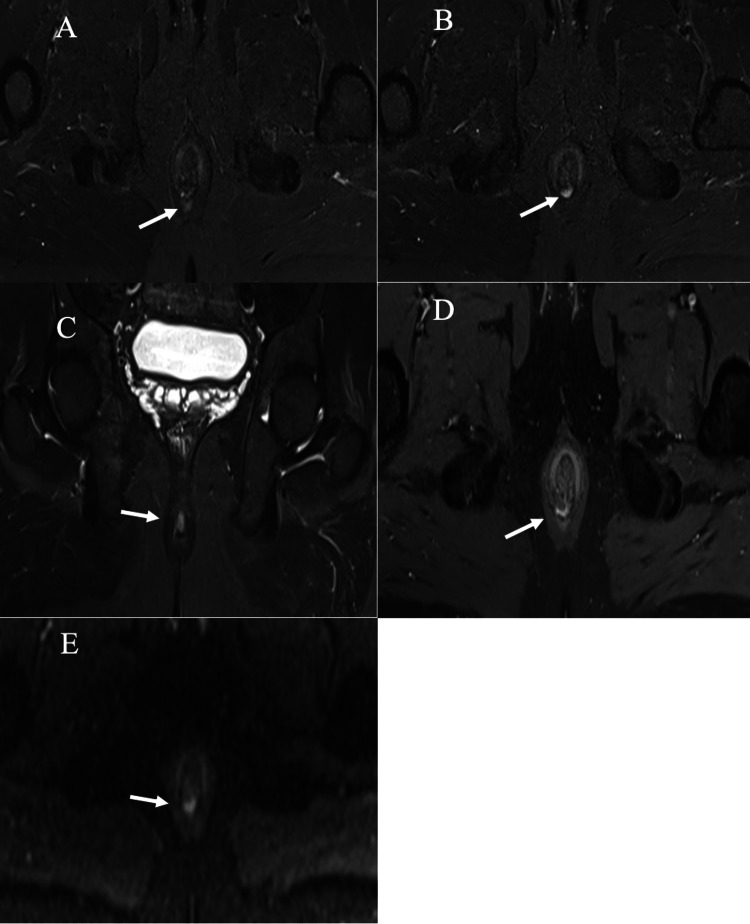
Grade 1 simple linear intersphincteric fistula: (A-B) Axial STIR images showing a simple linear intersphincteric fistula (A) with an internal opening at the 6 o’clock position (B). (C-D) Coronal STIR and axial post-contrast-enhanced fat-suppressed T1 images showing a simple linear intersphincteric fistula. (E) Axial DWI showing restricted diffusion on DWI. STIR: short tau inversion recovery; DWI: diffusion-weighted imaging

**Figure 2:**
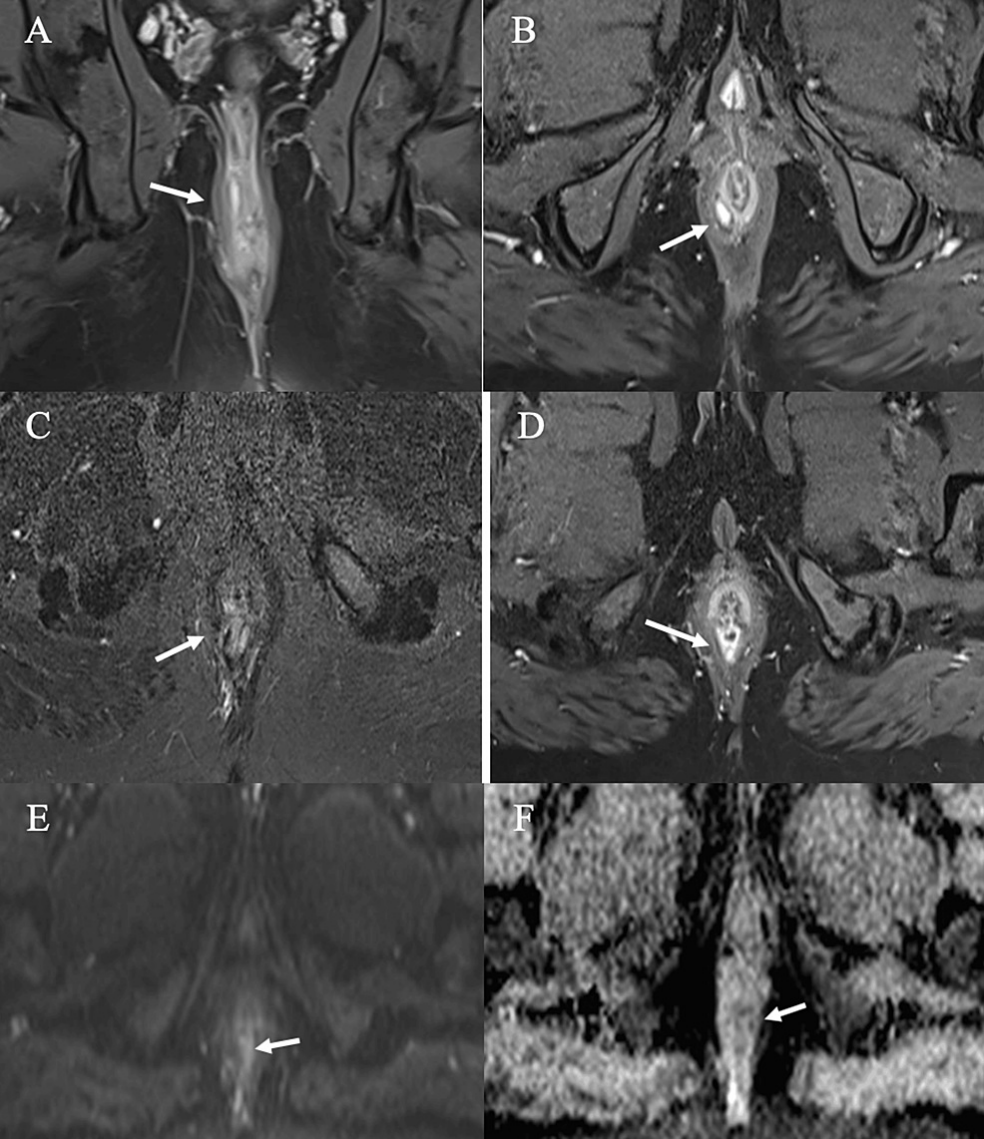
Grade 2 intersphincteric fistula with abscess. (A-B) Coronal STIR and axial post-contrast fat-suppressed T1-WI showing an intersphincteric fistula. (C-F) Axial STIR, post-contrast fat-suppressed T1, DWI, and ADC, respectively, depicting mild collection showing a hyperintense signal on T2 STIR (C), peripheral rim enhancement on the post-contrast image (D), and restricted diffusion DWI (E) with a drop on ADC map (F). DWI: diffusion-weighted imaging, STIR: short tau inversion recovery; ADC: apparent diffusion coefficient

**Figure 3:**
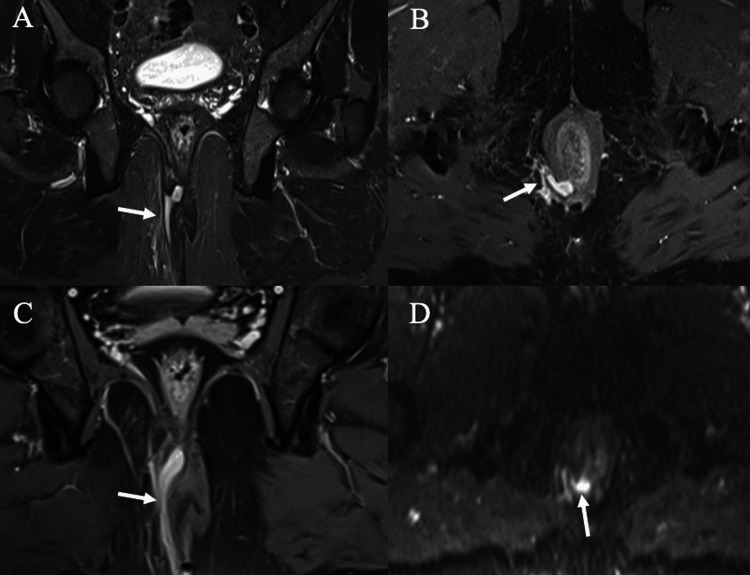
Grade 3 trans-sphincteric fistula. (A) Coronal STIR image showing right trans-sphincteric fistula. (B-C) Axial and coronal post-contrast-enhanced fat-suppressed T1 images, respectively, showing right trans-sphincteric fistula. (D) Axial DWI showing restricted diffusion on DWI. DWI: diffusion-weighted imaging, STIR: short tau inversion recovery

**Figure 4:**
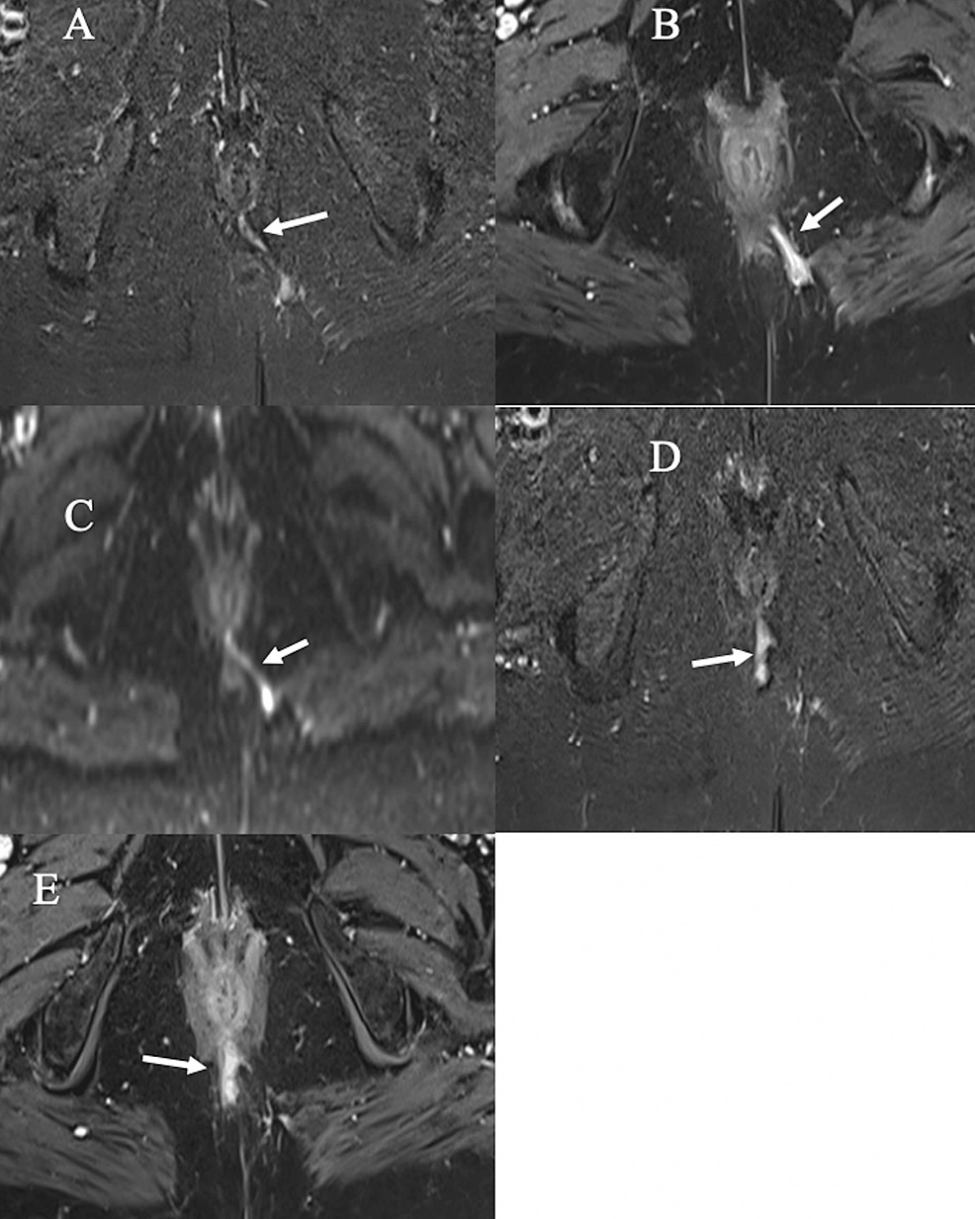
Grade 4 trans-sphincteric fistula with secondary ramification. (A-C) Axial STIR, post-contrast fat-suppressed T1-WI and DWI, respectively, showing grade 4 trans-sphincteric fistula crossing the external sphincter with a suspicious internal opening at the 6 o’clock position with restricted diffusion on DWI. (D-E) Axial STIR and post-contrast fat-suppressed T1, respectively, show secondary ramification coursing posteriorly. DWI: diffusion-weighted imaging, STIR: short tau inversion recovery

**Figure 5:**
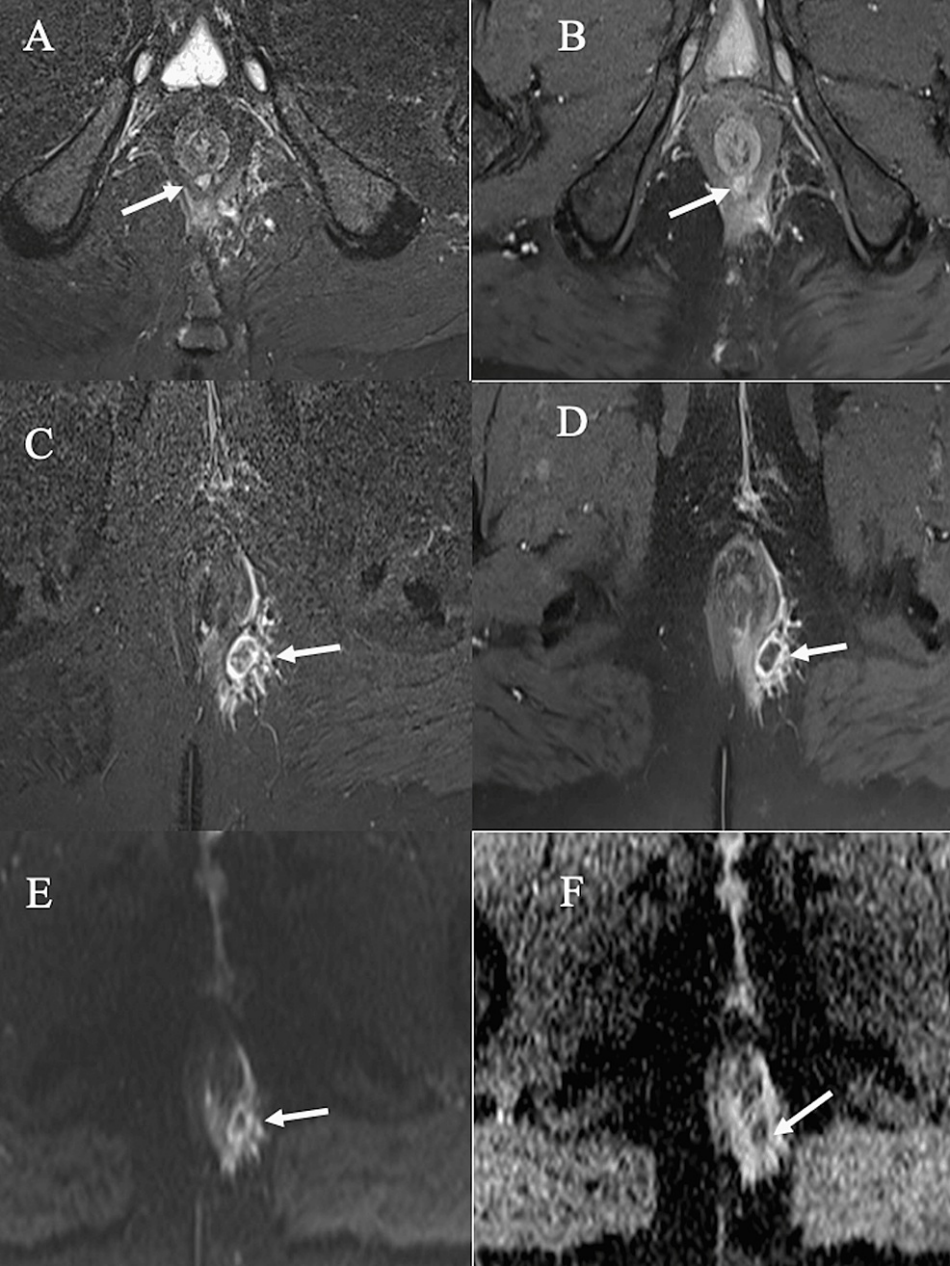
Grade 4 trans-sphincteric fistula with an abscess. (A-B) Axial STIR and post-contrast fat-suppressed T1-WI, respectively, showing grade 4 trans-sphincteric fistula crossing the external sphincter at the 5 o’clock position. (C-F) Axial STIR, post-contrast fat-suppressed T1, DWI, and ADC map, respectively, depicting abscess showing high signal intensity on STIR, peripheral rim enhancement on post-contrast fat-suppressed T1-WI and restricted diffusion in DWI and ADC map. DWI: diffusion-weighted imaging, STIR: short tau inversion recovery; ADC: apparent diffusion coefficient

**Figure 6:**
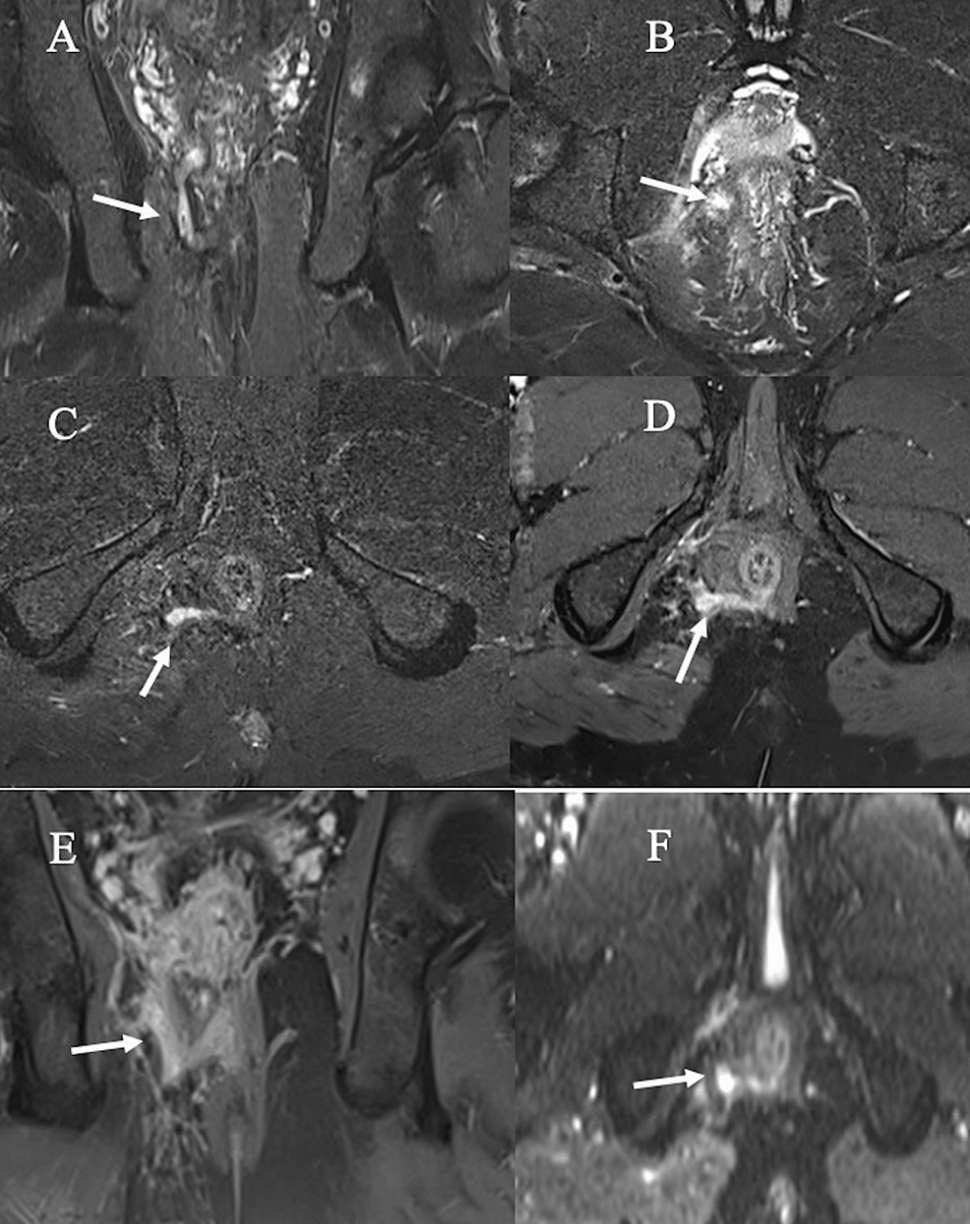
Grade 5 supralevator fistula: (A-B) Coronal STIR and axial post-contrast fat-suppressed T1-WI showing Grade 5 supralevator fistula. (C-F) Axial STIR, axial, coronal post-contrast fat-suppressed T1-WI, and DWI, respectively, showing trans-sphincteric medial ramification, which is crossing external sphincter with restricted diffusion on DWI. DWI: diffusion-weighted imaging, STIR: short tau inversion recovery

**Figure 7:**
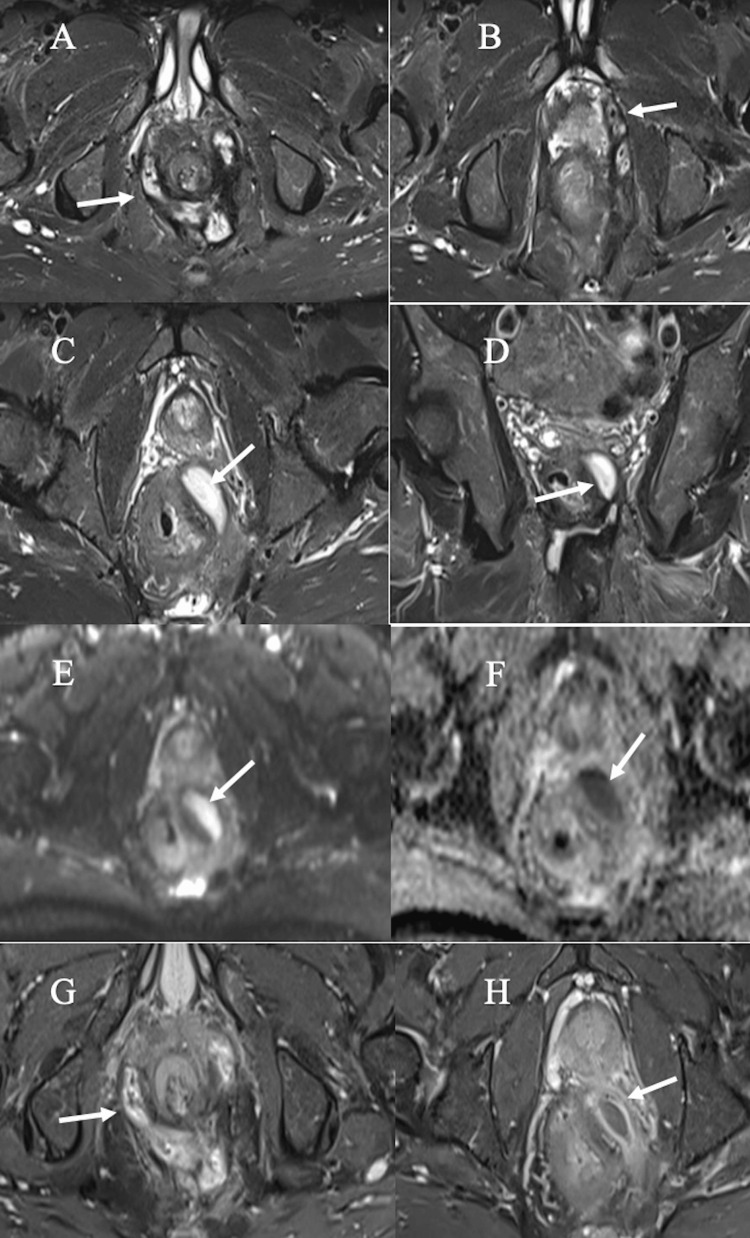
Horseshoe fistula. (A-C) Axial STIR images showing horseshoe fistula in the intersphincteric space (A) with multiple small abscesses in the intersphincteric space (B) and a well-defined abscess in left peri-rectal space (C). Coronal STIR image showing abscess in the left peri-rectal space (D). Axial DWI and ADC images showing restricted diffusion on DWI and ADC (E-F). Post-contrast fat-suppressed T1-WI showing horseshoe fistula (G) and peripheral rim enhancement in abscess (H). DWI: diffusion-weighted imaging, STIR: short tau inversion recovery; ADC: apparent diffusion coefficient

A few cases from our study as classified according to the St. James's University Hospital classification system (Figures 1-7).

## Conclusions

In our study, we concluded that the combination of DWI and T2 STIR is equivalent to post-contrast MRI in the identification and characterization of primary as well as complicated fistulous disease. The combination of DWI and T2 STIR is superior to DWI alone in the evaluation of perianal fistula. DWI is superior to T2 STIR in differentiating perianal inflammation with abscess from perianal inflammation without abscess and can be used as an alternative to post-contrast fat-suppressed T1-WI in the detection of perianal abscesses. DWI is also comparable to the post-contrast fat-suppressed T1 sequence in the assessment of disease activity. For detecting other fistula parameters, such as internal opening, secondary extensions, horseshoeing, etc., T2 STIR alone had superior diagnostic performance than DWI; however, their combination was superior to either of them and showed comparable performance to post-contrast images.

Ultimately, we believe that DWI offers several advantages as it can obviate the need for contrast administration and can be implemented easily at a commercial level with easy acquisition and post-data processing. However, when used alone, DWI has very low sensitivity for most fistula parameters. Therefore, it is best to use DWI as an adjunct to T2 STIR in the classification and characterization of perianal fistula, and the combination of DWI and T2 STIR can replace post-contrast fat-suppressed T1-WI, especially in cases where contrast administration is not feasible or contraindicated.
